# Practical Guidance for the Management of Adverse Events in Patients with KRAS^G12C^-Mutated Non-Small Cell Lung Cancer Receiving Adagrasib

**DOI:** 10.1093/oncolo/oyad051

**Published:** 2023-03-09

**Authors:** Jun Zhang, Melissa Johnson, Minal Barve, Lyudmila Bazhenova, Marybeth McCarthy, Rowena Schwartz, Elise Horvath-Walsh, Karen Velastegui, Chunlin Qian, Alexander Spira

**Affiliations:** Division of Medical Oncology, Department of Internal Medicine, Department of Cancer Biology, University of Kansas Medical Center, Kansas City, KS, USA; Sarah Cannon Research Institute Tennessee Oncology, Nashville, TN, USA; Mary Crowley Cancer Research, Dallas, TX, USA; UC San Diego Moores Cancer Center, La Jolla, CA, USA; Thoracic Oncology Clinical Trials, Henry Ford Cancer Institute, Detroit, MI, USA; University of Cincinnati, Cincinnati, OH, USA; Mirati Therapeutics, Inc., San Diego, CA, USA; Mirati Therapeutics, Inc., San Diego, CA, USA; Mirati Therapeutics, Inc., San Diego, CA, USA; Virginia Cancer Specialists, Fairfax, VA, USA; NEXT Oncology Virginia, US Oncology Research, The Woodlands, TX, USA

**Keywords:** adverse events, management, adagrasib, KRAS^G12C^ mutation, non-small cell lung cancer, safety

## Abstract

Adagrasib (MRTX849) is a KRAS^G12C^ inhibitor with favorable properties, including long half-life (23 h), dose-dependent pharmacokinetics, and central nervous system (CNS) penetration. As of September 1, 2022, a total of 853 patients with KRAS^G12C^-mutated solid tumors, including patients with CNS metastases, had received adagrasib (monotherapy or in combination). Adagrasib-related treatment-related adverse events (TRAEs) are generally mild to moderate in severity, start early in treatment, resolve quickly with appropriate intervention, and result in a low rate of treatment discontinuation. Common TRAEs seen in clinical trials included gastrointestinal-related toxicities (diarrhea, nausea, and vomiting); hepatic toxicities (increased alanine aminotransferase/aspartate aminotransferase) and fatigue, which can be managed through dose modifications, dietary modifications, concomitant medications (such as anti-diarrheals and anti-emetics/anti-nauseants) and the monitoring of liver enzymes and electrolytes. To manage common TRAEs effectively, it is imperative that clinicians are informed, and patients are fully counseled on management recommendations at treatment initiation. In this review, we provide practical guidance on the management of adagrasib TRAEs and discuss some best practices for patient and caregiver counseling to facilitate optimal outcomes for patients. Safety and tolerability data from the phase II cohort of the KRYSTAL-1 study will be reviewed and presented with practical management recommendations based on our experience as clinical investigators.

## Teaser

This review provides practical guidance on the management of adverse events related to adagrasib treatment and best practices for patient and caregiver counseling to facilitate optimal outcomes for patients.

**Implications for Practice**

This article provides recommendations for optimization of care and practical management of the most common adverse events seen with adagrasib in clinical trials in patients with KRAS^G12C^-mutated non-small cell lung cancer. The most common treatment-related adverse events (TRAEs) include gastrointestinal toxicities, hepatic toxicities, and fatigue. TRAEs are largely mild to moderate in severity and can be managed with appropriate strategies, including dose modification, dietary modifications, concomitant medications, and monitoring. Best practices for patient counseling are also discussed to ensure optimal outcomes for patients.

## Introduction

Kirsten rat sarcoma viral oncogene homolog (KRAS) is a membrane-associated guanosine triphosphatase that has the highest mutation rate of all known oncogenes across human cancers.^1^ KRAS mutations occur in approximately 25% of non-small cell lung cancer (NSCLC) cases^2^ and the KRAS^G12C^ point mutation in codon 12 occurs in approximately 14% of lung adenocarcinomas.^3^ Historically, KRAS mutations have been considered to be undruggable; however, this has changed with recent advances in the ability to target KRAS^G12C^ mutations.^1^

Adagrasib (MRTX849) is a potent, orally available, small-molecule covalent inhibitor of KRAS^G12C^, that irreversibly and selectively binds KRAS^G12C^ in its inactive, GDP-bound state.^4,5^ Adagrasib has favorable properties, including a long half-life (23 hours), dose-dependent pharmacokinetics, and central nervous system (CNS) penetration.^4,6^ Clinical activity of adagrasib has been seen in patients with KRAS^G12C^-mutated solid tumors, including NSCLC, colorectal, pancreatic, and biliary tract cancers.^7-10^ Data from the registrational Phase II cohort (*N* = 116; cohort A) of the KRYSTAL-1 study (NCT03785249) demonstrated an objective response rate (ORR) of 43% in patients with KRAS^G12C^-mutated NSCLC.^7^ Adagrasib has been shown to penetrate into the cerebrospinal fluid, resulting in tumor regression and extended survival in multiple preclinical CNS metastases models.^11^ Furthermore, clinical activity was also observed in patients with NSCLC and both treated, stable CNS metastases and active, untreated CNS metastases.^7,12^ In addition, adagrasib has demonstrated a manageable safety profile;^7,13^ further details and additional safety analyses are provided here. Based on these data, adagrasib was granted breakthrough status and was recently approved by the U.S. Food and Drug Administration for the treatment of NSCLC harboring a KRAS^G12C^ mutation who have received at least one prior systemic therapy. On December 21, 2022, adagrasib was included in the NCCN Clinical Practice Guidelines in Oncology (NCCN Guidelines) Non-Small Cell Lung Cancer with a category 2A recommendation as subsequent therapy for advanced NSCLC with a KRAS^G12C^ mutation.^14^ In addition, the adagrasib marketing application is under review by the European Medicines Agency.

Here we provide practical guidance on the management of treatment-related adverse events (TRAEs) most commonly associated with adagrasib based on first-hand clinical investigator experience. In addition, we discuss best practices for AE management and patient counseling to ensure optimal outcomes for patients at initiation of, and during, adagrasib treatment. AEs associated with adagrasib are generally mild to moderate in severity;^7,13^ however, it is essential that AEs are proactively and appropriately managed, and that patients receive adequate counseling and education.

## Clinical Trial Experience of Adverse Events Patterns and Management

### KRYSTAL-1 Cohort A Patient Demographics

The phase II cohort of the KRYSTAL-1 study (cohort A) evaluated adagrasib as monotherapy in patients with previously treated KRAS^G12C^-mutated NSCLC (*N* = 116). The updated data cut-off for this cohort was October 15, 2021 with a median follow-up of 12.9 months (95% confidence interval [CI] 11.8-13.5) and median duration of treatment of 5.7 months (range 0-19.6). The median age of patients in this trial was 64 years at the beginning of treatment (range 25-89), 96% were current or former smokers, and 84% had an Eastern Cooperative Oncology Group performance status score of 1. All patients (100%) had received previous platinum-based chemotherapy for NSCLC, and 114 patients (98%) had received both platinum-based chemotherapy and an immune checkpoint inhibitor; the median number of prior systemic therapies was 2.^7^

### KRYSTAL-1 Cohort A Summary of TRAEs

**Figure 1.**
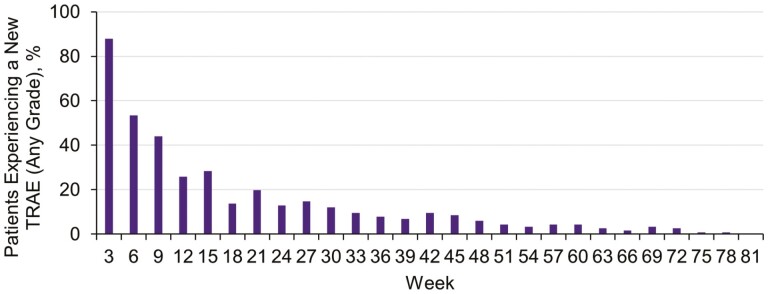
TRAE onset (cycle length 3 weeks). TRAE, treatment-related adverse event.

TRAEs (attributed by the investigators) of any grade occurred in 97% of patients and, of these, 18% were grade 1 and 35% were grade 2. Overall, 41% of patients experienced a grade 3 TRAE and 3% experienced a grade 4 TRAE (Table 1). The most common grade ≥3 TRAEs were increases in serum lipase (6%) and anemia (5%); there was one case of acute pancreatitis. The most common TRAEs were gastrointestinal (GI)-related events (diarrhea, nausea, or vomiting), fatigue, and increased alanine aminotransferase (ALT)/aspartate aminotransferase (AST); the majority of these TRAEs were grades 1-2. New onset TRAEs generally occurred early in treatment (Fig. 1), with >92% occurring within the first 9 weeks.

**Table 1.** Maximum grade TRAEs reported in cohort A of the KRYSTAL-1 study.

Event	Any grade	Grade 1	Grade 2	Grade 3	Grade 4
Any TRAE, *n* (%)	113 (97%)	21 (18%)	40 (35%)	47 (41%)	3 (3%)
Most common TRAEs, *n* (%)^a^					
Diarrhea	73 (63%)	56 (48%)	16 (14%)	1 (1%)	0
Nausea	72 (62%)	44 (38%)	23 (20%)	5 (4%)	0
Vomiting	55 (47%)	42 (36%)	12 (10%)	1 (1%)	0
Fatigue	47 (41%)	19 (16%)	23 (20%)	5 (4%)	0
ALT increase	32 (28%)	16 (14%)	11 (10%)	4 (3%)	1 (1%)
Blood creatinine increase	30 (26%)	21 (18%)	8 (7%)	1 (1%)	0
AST increase	29 (25%)	15 (13%)	10 (9%)	4 (3%)	0
Decreased appetite	28 (24%)	10 (9%)	14 (12%)	4 (3%)	0
Anemia	21 (18%)	6 (5%)	9 (8%)	6 (5%)	0
Amylase increase	20 (17%)	11 (10%)	8 (7%)	1 (1%)	0
Electrocardiogram QTc prolongation	19 (16%)	10 (9%)	4 (3%)	5 (4%)	0
Lipase increase	16 (14%)	2 (2%)	7 (6%)	7 (6%)	0
Blood alkaline phosphatase increase	12 (10%)	3 (3%)	4 (3%)	5 (4%)	0
Dysgeusia	12 (10%)	8 (7%)	4 (3%)	0	0
Hyponatremia	12 (10%)	4 (3%)	3 (3%)	5 (4%)	0
Edema peripheral	12 (10%)	10 (9%)	2 (2%)	0	0
Serious TRAE	20 (17%)	—	—	—	—

^a^Occurring in >10% of patients (any grade). Two grade 5 TRAEs occurred; one patient had cardiac failure (patient had a medical history of pericardial effusion but cardiac failure was not due to tamponade), and one patient had pulmonary hemorrhage (patient had a hilar lesion that infiltrated the pulmonary artery and led to a vascular rupture).

Adapted from Supplementary Table S3 by Jänne et al^7^, with permission from Massachusetts Medical Society.

Abbreviations: ALT, alanine aminotransferase; AST, aspartate aminotransferase; QTc, QT corrected interval; TRAE, treatment-related adverse event.

### TRAEs Leading to Dose Reductions, Interruptions, and Discontinuations

TRAEs led to dose reductions in 60 patients (52%) and dose interruptions in 71 patients (61%). The most common reasons were GI-related (diarrhea, nausea, or vomiting), hepatic (ALT/AST increase), and fatigue (Table 2). The initial dose reduction was from adagrasib 600 mg BID to adagrasib 400 mg BID, with an additional reduction to adagrasib 600 mg once daily, if needed. Among patients with a tumor response (*n* = 48), 44% of patients maintained a dose of 600 mg BID all or most of the time (>50% of treatment duration), as defined by timing of first dose reduction, while 42% of patients received 400 mg BID, 10% received 600 mg once daily, and 2% received 200 mg BID for the majority of treatment (>50% of treatment duration).^7^ Overall, 8 patients (7%) discontinued adagrasib due to TRAEs. Evaluations are ongoing with a lower dose of adagrasib 400 mg BID in clinical trials.

**Table 2.** TRAEs leading to dose reduction, interruption, or discontinuation.^a^

Event	Adagrasib monotherapy (N=116)
**TRAEs leading to dose reduction, *n* (%)**	60 (52%)
GI-related TRAEs^b^	23 (20%)
ALT increase	12 (10%)
Fatigue	11 (9%)
AST increase	7 (6%)
**TRAEs leading to dose interruption, *n* (%)**	71 (61%)
GI-related TRAEs^b^	26 (22%)
Fatigue	14 (12%)
ALT increase	11 (9%)
AST increase	10 (9%)
**TRAEs leading to discontinuation, *n* (%)**	8 (7%)
Ejection fraction decrease	2 (2%)
Transaminase increase	1 (1%)
Hypotension	1 (1%)
Pyrexia	1 (1%)
Unknown	3 (3%)

^a^Dose reductions and interruptions shown for only the most commonly occurring TRAEs (GI related, ALT/AST increase, and fatigue).

^b^Mostly diarrhea, nausea, or vomiting. Some patients experienced abdominal pain (*n* = 1), dyspepsia (*n* = 1), or pancreatitis (*n* = 1) that led to dose reductions, and abdominal pain (*n* = 2), abdominal distension (*n* = 1), or pancreatitis (*n* = 1) that led to dose interruptions.

Adapted from Jänne PA et al^7^, with permission from Massachusetts Medical Society (the full list is available in Supplementary Table S4).

Abbreviations: ALT, alanine aminotransferase; AST, aspartate aminotransferase; GI, gastrointestinal; TRAE, treatment-related adverse event.

## Adverse Event Management Recommendations

Patients and caregivers should be fully informed on ­adagrasib-related AEs prior to initiation of therapy. Coordination of care with healthcare providers is essential to assure that patients recognize toxicities and manage adverse effects appropriately since even Grade 2 AEs can be unpleasant and impact activities of daily living (Supplementary Table S1). In instances where supportive care measures are not sufficient and dose interruption or reduction is required, it is important that dose modifications are introduced at the appropriate time to quickly address the toxicity.

### Clinical Management of Common Adverse Events

#### GI Toxicity: Diarrhea, Nausea, Vomiting, Decreased Appetite

Diarrhea, nausea, and vomiting were the most commonly occurring AEs seen with adagrasib in cohort A of the KRYSTAL-1 study, occurring in 63%, 62%, and 47% of patients, respectively; decreased appetite also occurred in 24% of patients. Median time to onset for diarrhea, nausea, and vomiting events was 8 days (interquartile range [IQR] 2-28), 7 days (IQR 1-15), and 8 days (IQR 2-36), respectively (Supplementary Fig. S1). Median time from onset to resolution was 14 days (IQR 6-39), 21 days (IQR 10-56), and 5 days (IQR 2-24), respectively.

GI-related TRAEs were manageable with dose reductions/interruptions and/or use of supportive concomitant medications (including treatment and prophylaxis as needed), with overall use of anti-diarrheals in 48% and anti-emetics/anti-nauseants in 87% of patients. It should be noted that of the diarrhea and nausea cases that resolved, the majority were managed using supportive concomitant medications, whereas only a small minority of patients required a dose reduction or interruption due to diarrhea or nausea. Some clinical trial investigators chose to pre-dose patients who were experiencing GI-related AEs with both an anti-diarrheal and anti-emetic ~30 minutes prior to taking adagrasib throughout their treatment; patients were also given very small quantities of crackers or yogurt either directly prior to dosing or with dosing. In addition, some patients required regular IV hydration during initial cycles of the trial. It should be noted that all safety data for adagrasib to date relate to the capsule formulation, which is administered in a fasted state. After additional formulation development, all adagrasib clinical trials have now switched to the tablet formulation. Data in normal healthy volunteers with administration of single-dose adagrasib demonstrated lower incidence of diarrhea, nausea, and vomiting with the tablet versus capsule formulation (19.4% vs 35.1%, 13.9% vs 32.4%, and 0% vs 2.7%, respectively) and lower incidence for the tablet fed versus fasted conditions (12.5% vs 47.4%, 6.3% vs 36.8%, and 0% vs 21.1%, respectively) (Mirati data on file). The ability to take adagrasib as a tablet with food may help to manage GI-related adverse events.

#### GI Toxicity: Diarrhea

Management strategies to minimize diarrhea include dietary modifications such as eating small meals and avoiding greasy or spicy food, milk, caffeine, and alcohol. If diarrhea develops, patients may benefit from the B-R-A-T diet (bananas, rice, apple sauce, toast/decaffeinated tea) and maintaining fluids (6-8 large glasses of water, clear liquids, or soup per day). Medications such as diphenoxylate-atropine may be used to treat diarrhea. Oral administration of loperamide at low doses can be used; however, loperamide should be used with caution in certain patients, including those with underlying bradycardia, congestive heart failure (CHF), or prolonged QTc due to the risk of Torsades de Pointes (Table 3). Potassium and magnesium levels should be evaluated in patients with diarrhea to ensure they are maintained in appropriate range (Table 4). Dose modification guidance for diarrhea (per adagrasib clinical study protocols) is shown in Table 5.

**Table 3.** Recommendations for concomitant medications to be avoided.

Class	Examples of medications/substances to avoid	Potential effect
QT-prolonging medications	Azithromycin, chlorpromazine, ciprofloxacin, citalopram, clarithromycin, erythromycin, escitalopram, fluconazole, gatifloxacin, levofloxacin, loperamide^a^, moxifloxacin, ondansetron^b^	Effect of co-administering medicinal products known to prolong the QTc interval with adagrasib is unknown When not feasible to avoid concomitant administration of such drugs, conduct periodic ECG monitoring
CYP3A substrates with narrow therapeutic index	Alprazolam, cyclosporine, fentanyl, lovastatin, naloxegol, rivaroxaban, simvastatin	Increased exposure of CYP3A substrates, potentially increasing risk of SAEs associated with concomitant medication
Strong inducers of CYP3A	Carbamazepine, enzalutamide, rifampin, St. John’s wort	Decreased exposure to adagrasib, potentially reducing efficacy
CYP2D6 substrates with narrow therapeutic index^c^	Thioridazine, pimozide	Increased exposure of CYP2D6 substrate, potentially increasing risk of toxicities associated with concomitant medication
CYP2C9 substrates with narrow therapeutic index^c^	Warfarin^d^	Increased exposure of CYP2C9 substrate, potentially increasing risk of toxicities associated with concomitant medication
Substrates of P-gp	Digoxin	Increased exposure of P-gp substrate, potentially increasing risk of toxicities associated with concomitant medication

Note: this is not a comprehensive list of medications to avoid.

^a^Oral administration of loperamide at 0.5-1 mg per dose, up to 3 mg daily in patients without underlying bradycardia, congestive heart failure, or congenital long QT syndrome.

^b^Oral administration of ondansetron at doses up to 4 mg every 6 hours, with a maximum total daily dose of 16 mg, in patients without underlying bradycardia, congestive heart failure, or congenital long QT syndrome.

^c^If alternative treatments cannot be used, consider a dose reduction of the concomitant drug.

^d^If warfarin is co-administered, conduct additional international normalized ratio monitoring.

Abbreviations: BCRP, breast cancer resistance protein; CYP, cytochrome P450 enzyme; P-gp, P-glycoprotein 1; QT, QT interval; SAE, serious adverse event.

**Table 4.** Management strategies for common TRAEs.

TRAE	Management strategies
Diarrhea	Diarrhea prevention • Dietary modifications (eg, eating small meals and avoiding greasy or spicy food, milk, caffeine, and alcohol) Diarrhea management • B-R-A-T diet (bananas, rice, apple sauce, toast/decaffeinated tea) • Maintaining of fluids (6-8 large glasses of water, clear liquids, or soup per day) • Treatment with anti-diarrheals, such as diphenoxylate-atropine • Treatment with loperamide orally at low doses for patients without underlying bradycardia, CHF, or congenital long QT syndrome due to risk of Torsades de Pointes ◦ Monitor potassium and magnesium levels and supplement if below the reference range ◦ Perform regular ECGs • Dose modifications (Table 5)
Nausea and vomiting	• Administering the tablet formulation of adagrasib with food • Eating smaller, more frequent meals • Maintaining liquids • Monitoring electrolytes and supplementing as required • Engagement of dietician • For chronic nausea/vomiting: anti-emetics appropriate for the patient; choice of anti-emetic should be determined by patient-specific factors, and tolerability of anti-emetic side effects ◦ Treatment with ondansetron up to 4 mg every 6 hours (maximum total daily dose of 16 mg) for patients without underlying bradycardia, CHF, or congenital long QT syndrome ◦ Monitor potassium and magnesium levels and supplement if below the reference range ◦ Perform regular ECGs • For acute nausea/vomiting: treatment with 5-HT3 receptor antagonists (including palonosetron) and a short course of dexamethasone, or NK1 receptor antagonists • For refractory nausea/vomiting: treatment with olanzapine • For patients who take medications for gastric acidity, consideration should be given around the concomitant use of proton pump inhibitors (PPIs) due to a potential decrease in exposure of adagrasib ◦ Antacids or H2 blockers may be used instead • Dose modifications (Table 5)
Fatigue	• Exercising (taking plenty of breaks) • Staying hydrated • Keeping a normal sleep routine • Assessing potential contributing factors • Treatment with medications such as methylphenidate and modafinil (may decrease appetite so patients should be monitored) • Dose modifications (Table 5)
ALT/AST/alkaline phosphatase increase	• Monitoring liver enzymes regularly during treatment (every month for 3 months if the patient does not experience abnormalities) • Evaluating contributing factors • For significant hepatotoxicity, treatment with glucocorticoids can be considered • Dose modifications (Table 5)
Electrocardiogram QTc prolongation	• Managing electrolyte deficiencies (eg, potassium, magnesium, etc.) • Baseline ECGs and additional monitoring as clinically indicated (including in patients with symptomatic QT prolongation) • Dose modifications (Table 5) • Further information source: Credible Meds (https://www.crediblemeds.org/everyone)
Blood creatinine increase	• Assessing volume status and applying a low threshold for fluid replacement • Monitoring blood creatinine levels regularly during treatment (every month for 3 months if the patient does not experience abnormalities) • Nonsteroidal anti-inflammatory drugs (such as ibuprofen for pain) should be avoided or used with caution when creatinine is elevated • Dose modifications (Table 5)

Abbreviations: ALT, alanine aminotransferase; AST, aspartate aminotransferase; ECG, electrocardiogram; QTc, QT corrected interval; TRAE, treatment-related adverse event.

**Table 5.** Suggested dose modifications.

AEs	AEs by grade	Guidance
**GI toxicities**	Grade 1/2 AEs	Interrupt/modify dose at healthcare provider and patient discretion
	Grade 3 nausea >72h Grade 3/4 vomiting >24h Grade 3 diarrhea >48h	Interrupt adagrasib until ≤ grade 1 or return to baseline, and decrease one dose level
	Symptomatic grade 3/4 amylase or lipase elevation	CT scan to rule in/out acute pancreatitis. Interrupt adagrasib until ≤ grade 1 or return to baseline, and may resume one dose level lower
	Other grade 3 AEs	Interrupt adagrasib until ≤ grade 1 or return to baseline, and may resume at same dose level or one dose level lower
	Grade 4 diarrhea Grade 3/4 pancreatitis Other grade 4 AEs	Discontinue adagrasib
**Hepatic toxicities**	Grade 1/2 AEs (not including grade 2 ALT/AST increase)	Interrupt/modify dose at healthcare provider and patient discretion
	Grade 2 ALT/AST increase	Decrease one dose level
	Grade 3 ALT/AST increase Grade 3 increased bilirubin ≤22 days	Interrupt adagrasib until ≤ grade 1 or return to baseline, and decrease one dose level
	Grade 4 ALT/AST increase Hy’s Law Case Grade 3 increased bilirubin >22 days	Discontinue adagrasib
	Other Grade 3/4 AEs	Interrupt adagrasib until ≤ grade 1 or return to baseline, and may resume at same dose level or one dose level lower
**Cardiac toxicities**	QTcF prolongation >500 ms and increase >60 ms on at least 2 ECGs ≤22 days	Interrupt adagrasib until ≤ grade 1 or return to baseline (<15 ms above baseline), check electrolytes, and decrease one or two dose levels
	QTcF prolongation >500 ms and increase >60 ms on at least 2 ECGs >22 days Decrease in LVEF ≥20% from baseline and below LLN Symptomatic left ventricular systolic dysfunction	Discontinue adagrasib
	Other grade 3/4 AEs	Interrupt adagrasib until ≤ grade 1 or return to baseline, and may resume at one dose level lower
Other toxicities	Grade 1/2 AEs	Interrupt/modify dose at healthcare provider and patient discretion
	Grade 3/4 fatigue or asthenia ≤8 days	Interrupt adagrasib until ≤ grade 1 or return to baseline, and may resume at same or lower dose level
	Grade 3/4 creatinine increased ≤22 days Other lab-based grade 3/4 AEs	Interrupt adagrasib until ≤ grade 1 or return to baseline, and may resume at same dose level or one dose level lower
	Grade 3/4 creatinine increased >22 days	Discontinue adagrasib
	Other grade 3 AEs	Interrupt adagrasib until ≤ grade 1 or return to baseline, and resume one or more dose levels lower
	Other grade 4 AEs	Interrupt adagrasib until ≤ grade 1 or return to baseline, and may resume at a lower dose level (only if not life-threatening and can be managed; otherwise permanently discontinue adagrasib)

Sequential dose reduction steps: 400 mg BID, 600 mg once daily (may be split into BID dosing for tolerability).

Abbreviations: AE, adverse event; ALT, alanine aminotransferase; AST, aspartate aminotransferase; ECG, electrocardiogram; GI, gastrointestinal; LLN, lower limit of normal; LVEF, left ventricular ejection fraction; QTcF, QT corrected interval by Fredericia.

#### GI Toxicity: Nausea and/or Vomiting

Nausea and/or vomiting typically occur early in treatment (commonly within an hour of dosing) and tend to diminish over time; administering the tablet formulation of adagrasib with food may help to prevent nausea and vomiting from occurring. Patients should ensure adequate oral intake (eg, by eating smaller, more frequent meals) and maintain fluids; electrolytes should be monitored and supplemented as required. The engagement of a dietician may be helpful to optimize both calories and oral intake. Chronic nausea and/or vomiting may be treated using anti-emetics appropriate for the patient; choice of anti-emetic should be determined by patient-­specific factors, and tolerability of anti-emetic side effects. For example, prochlorperazine may be associated with sedation that adversely affects the patient; dose reduction of prochlorperazine or use of lorazepam could be considered in this case. Since ondansetron is known to prolong QTc (Table 3), doses up to 4 mg every 6 hours (maximum total daily dose 16 mg) should only be given in patients without underlying bradycardia, CHF, or congenital long QT syndrome; potassium and magnesium levels should be obtained at the beginning of each cycle and supplemented if below the reference range, and regular ECGs should be performed. For acute nausea/vomiting, 5-HT3 receptor antagonists (including palonosetron) and a short course of dexamethasone can be given, or NK1 receptor antagonists (eg, aprepitant). Refractory nausea/vomiting can be treated with olanzapine. If a patient vomits after taking adagrasib, they should not take an additional dose, but wait until their next dose is due. Dose modification guidance for nausea and vomiting (per adagrasib clinical study protocols) is shown in Table 5.

Guidance should be given to patients who take medications for gastric acidity since adagrasib solubility is maximal at low pH. While 53% of patients in cohort A of the KRYSTAL-1 study were treated with proton pump inhibitors (PPIs), consideration should be given around the concomitant use of PPIs due to a potential decrease in exposure of adagrasib. Importantly, as PPIs are available over the counter, patients should be counseled to talk to healthcare providers prior to initiation of any medications for gastric symptoms. Alternatives, such as antacids or H2 blockers, may be used; 20% of patients were treated with H2 blockers in cohort A of the KRYSTAL-1 study. In addition, an acid buffering agent such as sucralfate may also be used.

#### Fatigue

In cohort A of the KRYSTAL-1 study, fatigue was reported as a TRAE in 41% of patients. Median time to onset was 15 days (IQR 8-36) and median time from onset to resolution was 29 days (IQR 15-61). Treatment-related fatigue led to dose reductions and interruptions in 11 patients (9%) and 14 patients (12%), respectively.

Symptoms of fatigue include chronic tiredness and impaired judgment. Management strategies for fatigue include exercise (taking plenty of breaks), staying hydrated, and keeping a normal sleep routine. Fatigue may be caused by many factors, including some of the symptoms already discussed; potential contributing factors (eg, electrolyte imbalance, reduced calorie intake, iron or B12 deficiencies, hypothyroidism, etc.) should be evaluated and management strategies implemented along with correcting other underlying abnormalities. In addition, medications such as methylphenidate and modafinil, which are used in the management of cancer-related fatigue, could be considered based on patient-specific considerations.^15^ Clinical investigators observed that medications to combat fatigue were rarely used during the KRYSTAL-1 study and counseling patients to take additional rest was often used instead. Dose modification guidance for fatigue (per adagrasib clinical study protocols) is shown in Table 5.

#### Hepatic: AST/ALT/Alkaline Phosphatase Increase

In the KRYSTAL-1 study, increased ALT and AST were reported as a TRAE in 28% and 25% of patients, respectively. Median time to onset of increased ALT and AST was 22 days (IQR 15-26) and 22 days (IQR 15-35), respectively, and median time from onset to resolution was 14 days (IQR 8-22) and 11 days (IQR 6-22), respectively. Treatment-related alkaline phosphatase increase occurred in 12 patients (10%); median time to onset was 25 days (IQR 18-46), and median time from onset to resolution was 12 days (IQR 8-20). Treatment-related increase in ALT, AST, and alkaline phosphatase led to relatively low rates of dose reductions and interruptions. There were no Hy’s Law cases (defined as ALT >3 times upper limit of normal [ULN] and total bilirubin >2 times ULN) reported to date in the adagrasib clinical development program.

Mild transaminitis is most commonly asymptomatic, therefore liver enzymes should be monitored regularly during treatment (every month for 3 months if the patient does not experience abnormalities). More frequent monitoring of liver function (including AST, ALT, alkaline phosphatase, and bilirubin) is advised for patients with transaminase increases. When transaminases are increased it is important to evaluate other potential contributing factors such as alcohol use, acetaminophen, other medications, and medical conditions. In the case of more significant hepatotoxicity, treatment with glucocorticoids can be considered. Dose modification guidance for hepatic toxicities (per adagrasib clinical study protocols) is shown in Table 5.

#### Blood Creatinine Elevation

Treatment-related blood creatinine increase occurred in 26% of patients in cohort A of the KRYSTAL-1 study. Median time to onset was 11 days (IQR 8-46) and median time from onset to resolution was 19 days (IQR 14-36). The increase led to dose reductions and interruptions in 1 patient (1%) and 7 patients (6%), respectively.

Blood creatinine increases may represent pre-renal or post-renal causes, so volume status should be assessed and a low threshold for fluid replacement should be applied. Blood creatinine levels should be monitored regularly during treatment (every month for 3 months if the patient does not experience abnormalities). Nonsteroidal anti-inflammatory drugs (such as ibuprofen for pain) should be avoided or used with caution when creatinine is elevated. Preclinical models have not supported evidence for nephrotoxicity due to adagrasib. It should be noted that mild blood creatinine increases may be artificial (eg, due to multidrug and toxin extrusion transporter 1 inhibition) and have no effect on the glomerular filtration rate.^16^ Dose modification guidance for blood creatinine elevation (per adagrasib clinical study protocols) is shown in Table 5.

#### Cardiac Toxicity: QT Prolongation

In cohort A of the KRYSTAL-1 study, treatment-related ECG QT prolongation occurred in 16% of patients. Median time to onset was 8 days (IQR 7-8) and median time from onset to resolution was 17 days (IQR 14-43). Treatment-related ECG QT prolongation led to a low rate of dose reductions and interruptions. The mean QTc (by Fredericia) interval change from baseline for adagrasib was 17.93 ms; no patient had any evidence of clinical arrhythmias. In general, QTc increases occurred once patients reached steady-state drug concentrations at 8 days but did not generally increase later in treatment. Patients with certain cardiac abnormalities, including a prolonged QTc interval >480 ms during the screening period or a family or medical history of congenital long QT syndrome, were excluded from the study.

A corrected QT interval above 500 ms or an increase of >60 ms from baseline is considered grade 3, which may pose a risk to the patient.^17,18^ QT prolongation is usually asymptomatic, but can cause syncope and ventricular arrhythmias, including Torsades de Pointes. Baseline ECGs and additional/routine assessments should be performed as clinically indicated (including in patients with symptomatic QT prolongation). Management strategies include correcting modifiable risk factors, such as electrolyte deficiencies (eg, potassium and magnesium) and additional monitoring (eg, regular electrocardiograms). Dose interruption along with cardiologist referral should be considered when QTc prolongation is grade ≥3; dose modification guidance for cardiac toxicities (per adagrasib clinical study protocols) is shown in Table 5.

#### Other TRAEs

In the KRYSTAL-1 study, treatment-related pneumonitis was reported in 5% of patients in cohort A, of which 3% were grades 1-2 and 2% were grade 3. Patients should be monitored for new or worsening respiratory symptoms indicative of pneumonitis (eg, dyspnea, cough, fever), and a CT scan should be considered. Since treatment-related pneumonitis cases in KRYSTAL-1 generally improved with treatment interruption, adagrasib should be withheld in patients with suspected pneumonitis and permanently discontinued if grade ≥3 and no other potential causes of pneumonitis can be identified. Prompt initiation of steroids and a pulmonology consult should be considered, especially for moderate and severe cases of pneumonitis.

Treatment-related skin hyperpigmentation was reported in 9% of patients in cohort A of the KRYSTAL-1 study;^7^ however, it is the opinion of the clinical investigators that this was largely under-reported as it may not be noticeable in patients with darker skin tones. For some patients, such as those with fair skin tones, skin hyperpigmentation can present as a bothersome orange pigment; therefore, it is important to counsel patients that this is a potential side effect of treatment with adagrasib.

### Recommended Frequency of Monitoring

The clinical investigators recommend monitoring liver function tests once per month for the first 3 months followed by a standard-of-care (SOC) frequency. Other blood tests, including blood counts, chemistry, amylase, and lipase should be monitored in accordance with SOC, or when clinically warranted. ECGs should be monitored prior to treatment and as early as 1 week after initiating treatment, then monitored as clinically indicated. More frequent ECG monitoring should be considered in patients with congestive heart failure, bradyarrhythmias, electrolyte abnormalities, and in patients who are unable to avoid concomitant medications that are known to prolong the QT interval.

## Concomitant Medications/Drug Interactions

There are some medications that should be avoided for patients taking adagrasib. Some examples are given in Table 3, and it is recommended that healthcare providers review all of the patient’s medications carefully for potential drug-drug interactions prior to initiation of adagrasib and routinely throughout therapy. As some medications that should be avoided may be used to treat AEs associated with adagrasib, it is paramount that providers are aware of these restrictions and alternative management strategies that can be adopted instead. Any patients who are given concomitant medications that could impact QTc should be monitored at baseline and at regular intervals. It is also important to counsel patients about medications that they should not take when being treated with adagrasib and discuss any potential drug interactions. Patients should be advised to report to their clinician any medications or herbal remedies they are taking/planning to take; many of these are over-the-counter products (eg, PPIs), which they may not realize have the potential for drug interactions.

## Patient Counseling

Counseling for patients on what side effects to expect, anticipated timing of side effects, and how they can be managed is very important. Effective counseling is a vital part of the successful and safe use of cancer therapies and helps patients to manage side effects without having to attend the clinic as regularly. In addition, studies have shown that providing information and support to patients is important for improving medication adherence; in fact, patient education can significantly improve compliance with medication across a broad range of conditions and disease severities.^19,20^ Verbal and written information should be combined, and patients should understand the importance of openly and rapidly communicating about their AEs. Patients should have supportive care medications readily available if they experience certain side effects, such as anti-emetics/anti-diarrheals if they experience GI toxicity. In other cases (although more rarely), patients may be prescribed prophylactic treatment to take in anticipation of AEs occurring. It is also helpful to highlight the use of oral fluids and foods that may prevent or help mitigate GI toxicity. Patients should be advised to tell their oncologist about any other medications they are taking for non-cancer related illnesses, to review for potential interactions. Patients should also be encouraged to keep dietary diaries to detect other medications, supplements, or food that may interact with adagrasib.

## Discussion

Historically, KRAS mutations were considered to be undruggable, and as such there have been limited therapy options for patients with advanced solid tumors harboring a KRAS^G12C^ mutation.^1^ However, advances have been made in the ability to target KRAS^G12C^-mutated solid tumors, including the approval of sotorasib in 2021 and adagrasib in 2022 for patients with metastatic KRAS^G12C^-mutated NSCLC.^21,22^ Adagrasib, a selective covalent inhibitor of KRAS^G12C^, has demonstrated manageable tolerability and promising clinical activity in patients with KRAS^G12C^-mutated NSCLC, colorectal, pancreatic, and biliary tract cancers, and in patients with KRAS^G12C^-mutated NSCLC and CNS metastases.^7-10,12^ The data presented in this manuscript are from patients with KRAS^G12C^-mutated NSCLC who were treated with adagrasib monotherapy; a similar safety profile for adagrasib has been seen across studies in other tumor types and combination regimens to date.^8,10,23^

AEs associated with adagrasib are generally mild to moderate in severity and can be managed with concomitant medications and dose modifications; 87% of patients were treated with anti-emetics and 48% received anti-diarrheals, but the discontinuation rate for adagrasib due to TRAEs was low (7%). The most common TRAEs are GI-related and should be managed with supportive care, anti-diarrheals, and anti-emetics. The ability to take adagrasib as a tablet with food may help to manage GI-related adverse events.

## Conclusion

Adagrasib-related TRAEs are generally mild and manageable with dose modifications (interruptions and/or reductions), concomitant medications (such as anti-diarrheals and anti-emetics), lifestyle changes, and individual patient counseling. Proactive toxicity management and patient education are key to assisting the patient in their treatment course.

## Supplementary Material

oyad051_suppl_Supplementary_Material

## Data Availability

At Mirati Therapeutics we are committed to patient care, advancing scientific understanding, and enabling the scientific community to learn from and build upon the research we have undertaken. To that end, we will honor legitimate requests for our clinical trial data from qualified researchers and investigators for conducting methodologically sound research. We will share clinical trial data, clinical study reports (CSRs), study protocols, and statistical analysis plans from clinical trials for which results have been posted on clinicaltrials.gov for products and indications approved by regulators in the US and/or EU. Sharing is subject to protection of patient privacy and respect for the patient’s informed consent. In general, data will be made available for specific requests approximately 24 months after clinical trial completion from our in-scope interventional trials. For additional information on proposals with regards to data sharing collaborations with Mirati, email us at medinfo@mirati.com.
