# Supplementary material for: Practical Guidance for the Management of Adverse Events in Patients with KRASG12C-Mutated Non-Small Cell Lung Cancer Receiving Adagrasib
Source: Oncologist. 2023 Mar 9;28(4):287–96. doi: 10.1093/oncolo/oyad051 (PMC10078892; doi:10.1093/oncolo/oyad051)
Supplement: oyad051_suppl_Supplementary_Material [file oyad051_suppl_supplementary_material.docx]

**Supplementary Material**

**Supplementary Table 1.** Adverse event grading according to the Common Terminology Criteria for Adverse Events (CTCAE) v5.0[1]

| **TRAE** | **Grade 1** | **Grade 2** | **Grade 3** | **Grade 4** | **Grade 5** |
| --- | --- | --- | --- | --- | --- |
| Diarrhea | Increase of <4 stools per day over baseline; mild increase in ostomy output compared with baseline | Increase of four to six stools per day over baseline; moderate increase in ostomy output compared with baseline; limiting instrumental ADL^a^ | Increase of seven or more stools per day over baseline; hospitalization indicated; severe increase in ostomy output compared with baseline; limiting self-care ADL^b^ | Life-threatening consequences; urgent intervention indicated | Death |
| Nausea | Loss of appetite without alteration in eating habits | Oral intake decreased without significant weight loss, dehydration or malnutrition | Inadequate oral caloric or fluid intake; tube feeding, TPN, or hospitalization indicated | NA | NA |
| Vomiting | Intervention not indicated | Outpatient IV hydration; medical intervention indicated | Tube feeding, TPN, or hospitalization indicated | Life-threatening consequences | Death |
| Fatigue | Fatigue relieved by rest | Fatigue not relieved by rest; limiting instrumental ADL^a^ | Fatigue not relieved by rest; limiting self-care ADL^b^ | NA | NA |
| AST increase | >ULN – 3.0 x ULN if baseline was normal; 1.5–3.0 x baseline if baseline was abnormal | >3.0–5.0 x ULN if baseline was normal; >3.0–5.0 x baseline if baseline was abnormal | >5.0–20.0 x ULN if baseline was normal; >5.0–20.0 x baseline if baseline was abnormal | >20.0 x ULN if baseline was normal; >20.0 x baseline if baseline was abnormal | NA |
| ALT increase | >ULN – 3.0 x ULN if baseline was normal; 1.5–3.0 x baseline if baseline was abnormal | >3.0–5.0 x ULN if baseline was normal; >3.0–5.0 x baseline if baseline was abnormal | >5.0–20.0 x ULN if baseline was normal; >5.0–20.0 x baseline if baseline was abnormal | >20.0 x ULN if baseline was normal; >20.0 x baseline if baseline was abnormal | NA |
| ALP increase | >ULN – 2.5 x ULN if baseline was normal; 2.0–2.5 x baseline if baseline was abnormal | >2.5–5.0 x ULN if baseline was normal; >3.0–5.0 x baseline if baseline was abnormal | >5.0–20.0 x ULN if baseline was normal; >5.0–20.0 x baseline if baseline was abnormal | >20.0 x ULN if baseline was normal; >20.0 x baseline if baseline was abnormal | NA |
| Electrocardiogram QTc prolongation | Average QTc 450–480 ms | Average QTc 481–500 ms | Average QTc ≥501 ms; >60 ms change from baseline | Torsade de pointes; polymorphic ventricular tachycardia; signs/symptoms of serious arrhythmia | NA |
| Blood creatinine increase | >ULN – 1.5 x ULN | >1.5–3.0 x baseline; >1.5–3.0 x ULN | >3.0 x baseline; >3.0–6.0 x ULN | >6.0 x ULN | NA |
| Skin hyperpigmentation | Hyperpigmentation covering <10% BSA; no psychosocial impact | Hypopigmentation or depigmentation covering >10% BSA; associated psychosocial impact | NA | NA | NA |

^a^Instrumental ADL refer to preparing meals, shopping for groceries or clothes, using the telephone, managing money, etc. ^b^Self-care ADL refer to bathing, dressing and undressing, feeding self, using the toilet, taking medications, and not bedridden

ADL, Activities of Daily Living; ALP, alkaline phosphatase; ALT, alanine aminotransferase; AST, aspartate aminotransferase; BSA, body surface area; IV, intravenous; NA, not applicable; QTc, QT corrected interval; TPN, total parenteral nutrition; ULN, upper limit of normal

Common Terminology Criteria for Adverse Events (CTCAE) Version 5.0 was originally published by the National Cancer Institute

**Supplementary Figure 1.** Time to Onset and Resolution of TRAEs


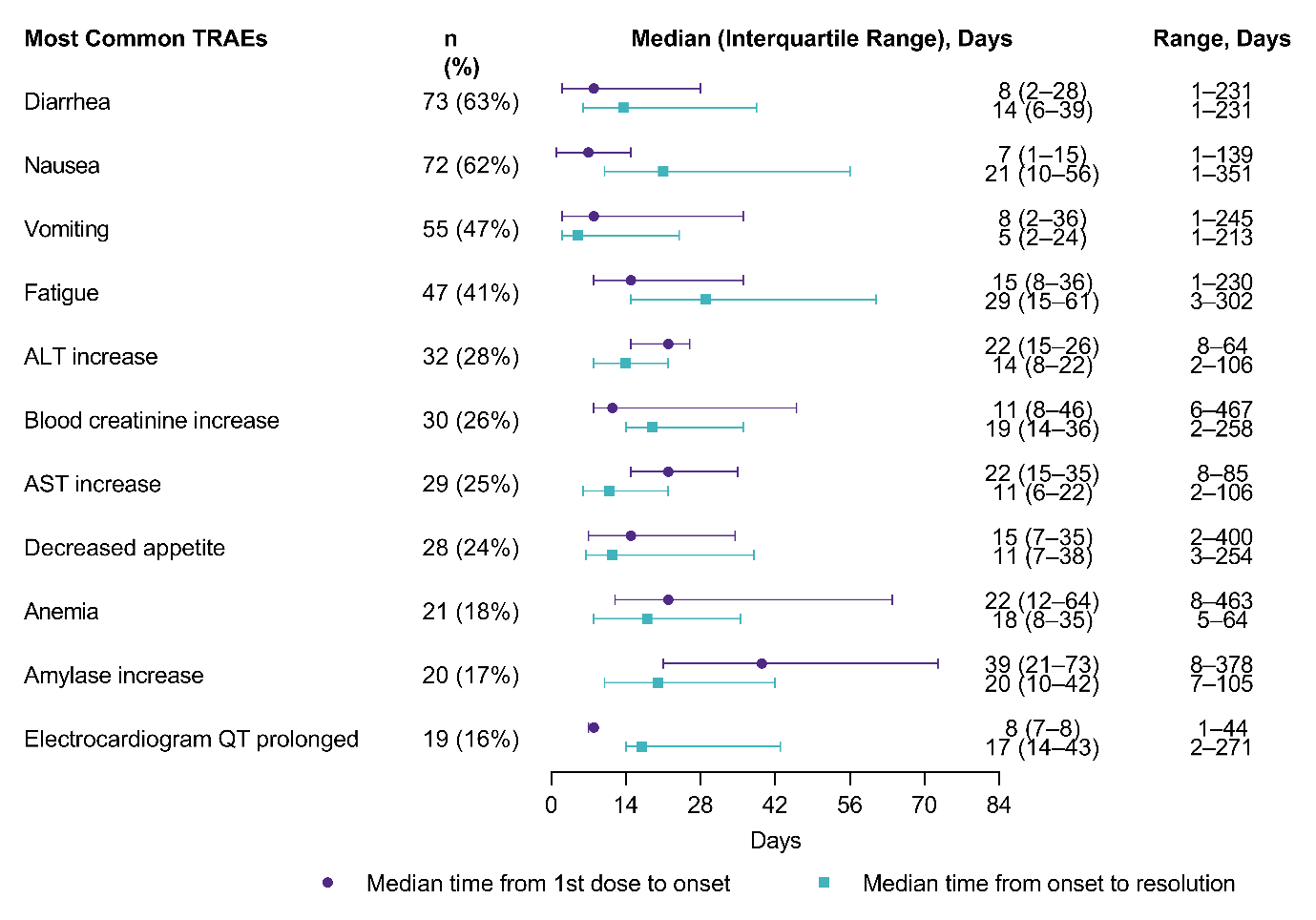


ALT, alanine aminotransferase; AST, aspartate aminotransferase; QT, QT interval; TRAE, treatment-related adverse event

**REFERENCES**

1. U.S. Department of Health and Human Services. Common Terminology Criteria for Adverse Events (CTCAE) version 5.0. <https://ctep.cancer.gov/protocoldevelopment/electronic_applications/docs/CTCAE_v5_Quick_Reference_5x7.pdf>.
